# Quantitative phase contrast imaging with a nonlocal angle-selective metasurface

**DOI:** 10.1038/s41467-022-34197-6

**Published:** 2022-12-21

**Authors:** Anqi Ji, Jung-Hwan Song, Qitong Li, Fenghao Xu, Ching-Ting Tsai, Richard C. Tiberio, Bianxiao Cui, Philippe Lalanne, Pieter G. Kik, David A. B. Miller, Mark L. Brongersma

**Affiliations:** 1 Geballe Laboratory for Advanced Materials, Stanford University, Stanford, CA 94305 USA; 2 Department of Chemistry, Stanford University, Stanford, CA 94305 USA; 3 Stanford Nano Shared Facilities, Stanford University, Stanford, CA 94305 USA; 4 LP2N, CNRS, University of Bordeaux, 33400 Talence, France; 5 CREOL, The College of Optics and Photonics, University of Central Florida, Orlando, FL 32816 USA; 6 Department of Electrical Engineering, Stanford University, Stanford, CA 94305 USA

**Keywords:** Metamaterials, Metamaterials

## Abstract

Phase contrast microscopy has played a central role in the development of modern biology, geology, and nanotechnology. It can visualize the structure of translucent objects that remains hidden in regular optical microscopes. The optical layout of a phase contrast microscope is based on a 4 *f* image processing setup and has essentially remained unchanged since its invention by Zernike in the early 1930s. Here, we propose a conceptually new approach to phase contrast imaging that harnesses the non-local optical response of a guided-mode-resonator metasurface. We highlight its benefits and demonstrate the imaging of various phase objects, including biological cells, polymeric nanostructures, and transparent metasurfaces. Our results showcase that the addition of this non-local metasurface to a conventional microscope enables quantitative phase contrast imaging with a 0.02π phase accuracy. At a high level, this work adds to the growing body of research aimed at the use of metasurfaces for analog optical computing.

## Abstract (web summary)

The authors present an approach to phase imaging by using the non-local optical response of a guided-moderesonator metasurface. They demonstrate that this metasurface can be added to a conventional microscope to enable quantitative phase contrast imaging.

## Introduction

Every day, we rely on a massive number of images to guide us in autonomous driving, medical diagnosis, surveillance, environmental monitoring, the Internet of Things, and scientific research. Empowered by the rapid advances in computer vision, we increasingly rely on computers to interpret these images and aid us in various decision-making processes. Deep learning^1^ with artificial neural networks and digital computers enables image analysis with a logic structure that aims to mimic how we think. As the ability of image processing systems is approaching that of humans, it has become possible to perform a wide range of complex image analysis tasks^2,3^. However, there are a variety of applications in/for which digital electronics cannot provide an end-all solution in interpreting images and optics can offer a helping hand. These include imaging tasks that require high-throughput, real-time, and low-power image processing or where critical information is lost in the action of taking an image.

Despite the increased demand for more sophisticated information and high-throughput, low-power processing, the architectures of man-made imaging systems have not dramatically evolved. We can learn from nature where the eyes of different animals have adapted to meet specific survival needs^4–6^. Recent research shows the possible benefits of co-designing the optics and electronics for image processing by offloading some of the ever-increasing burden on the digital electronics and offering new functionality. This has proven to be of particular value in the field of microscopy, where images are captured in highly-engineered environments that afford accurate control over the illumination optics, placement of samples, and alignment of imaging hardware. This has led to major breakthroughs in brightfield microscopy^7^, super-resolution imaging in fluorescence microscopy^8–10^, and lensless microscopy^11^.

**Fig. 1:**
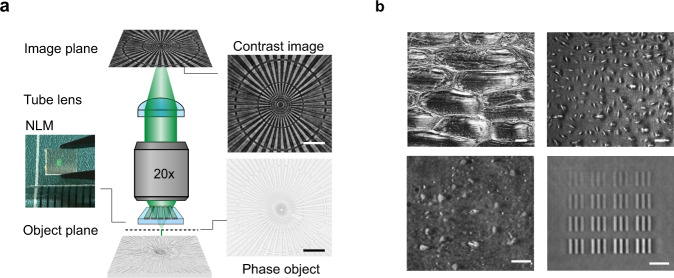
A nonlocal metasurface (NLM) patterned on a microscope slide enables phase contrast imaging. **a** A schematic showing how a classic bright field microscope can be transformed into a phase imaging setup simply by inserting an NLM on top of the phase object. The flat optical component, a commercial phase object and the corresponding phase contrast image are shown next to the microscope schematic. **b** NLM phase contrast images of onion cells (top left), human osteosarcoma cells (U-2 OS, top right), a dense array of exfoliated transparent hexagonal boron nitride (hBN) flakes (bottom left), and arrays of metasurface elements comprised of silicon nitride pillars with different diameters (bottom right). All scale bars in **a** and **b** correspond to 25 µm length.

Given these developments, it is of great interest to see whether co-design in microscopy can also make it possible to image the phase of light. The optical phase is arguably the most critical piece of information that is lost when capturing an image due to the nature of the photodetection process. Phase information can be retrieved using phase-contrast microscopy; however, modern phase-contrast microscopes still rely on the use of several optical components that need careful insertion into a microscope and require their precise alignment. Given the rapid developments in the field of flat optics^12–16^, it is worth exploring whether judiciously nanostructured, planar elements can provide innovative solutions. A recent wave of research has demonstrated that flat optics based on metasurfaces and photonic crystals are ideally suited to execute various image processing tasks^17–25^, including image differentiation or quantitative phase gradient retrieval that can be employed to extract valuable phase information. Metasurfaces have also already been integrated with image sensors to create compact systems that can extract more information, such as angles, angular momentum and polarization information, from the light field than traditional sensors^5,26–34^. Here, we show how phase contrast microscopy can be performed by inserting a nonlocal metasurface (NLM) between a phase object and the objective of a basic optical microscope (Fig. 1a). The NLM facilitates quantitative phase-contrast imaging by controlling the angle-dependent transmission amplitude and phase of incident light waves at a target illumination wavelength in a prescribed fashion. We discuss its design and use it to visualize various phase objects that are important in different fields of science and technology (Fig. 1b). We also show that, beyond imaging functions, the NLM affords highly accurate phase measurements of a large area in a single-shot image.

## Results

### Experiment-optical image processing scheme

**Fig. 2:**
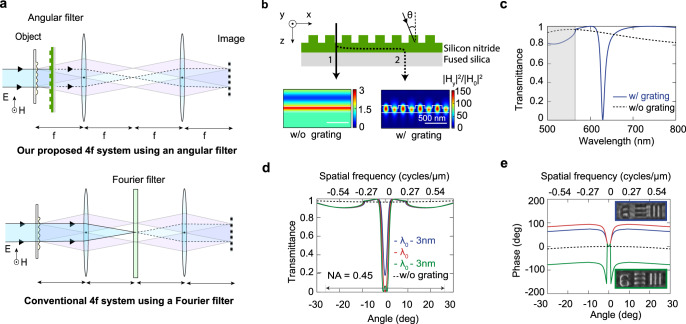
Optical image processing can be achieved with a NLM that serves as a designer angular filter. **a** Top: Our proposed phase contrast system utilizing an NLM-based angular filter. Bottom: A schematic of a conventional 4 *f* image processing system with a Fourier filter is shown for reference. **b** A cross sectional schematic of the NLM. Incident transverse magnetic (TM) polarized light can take a direct (1) and indirect, resonant pathway (2) through this optical element. The corresponding field distributions are shown underneath the schematic. **c** The simulated transmittance spectrum of our NLM at normal incidence shows a pronounced dip that results from destructive interference between the direct and indirect pathways on resonance. **d** Simulated angular transmittance spectra of the NLM on and near the resonant wavelength λ_0_. The case without the surface relief grating is shown for reference. **e** Simulated phase shift produced by the NLM for incident light at different angles. Insets show positive contrast images (blue box) and negative contrast images (green box) that were taken below and above the resonant wavelength, respectively.

Phase contrast microscopy allows for the visualization of translucent objects that are challenging to see in an ordinary light microscope as they hardly absorb or scatter light. It capitalizes on the fact that waves passing through different regions of a specimen acquire different propagation phases. To make these phase differences visible, a phase-contrast microscope first separates the undiffracted light (background) from the light that is diffracted by a specimen (foreground). These light waves are then manipulated in different ways so that they produce strong interference contrast when they are reunited in an image plane. The Nobel laureate Fritz Zernike proposed the use of a filter in the Fourier plane of a 4 f system for this purpose^35–37^. The 4 f system operates as a common path interferometer that synthesizes its own reference beam. An implementation in a 4 f image processing system is shown at the bottom of Fig. 2a. It is comprised of two lenses with a focal length *f* and a specialized Fourier filter, often called a phase plate. These components and the specimen are spaced in an equidistant fashion by a distance *f*. When the specimen is illuminated with a quasi-collimated beam, the different spatial frequencies in the phase distribution imparted by the specimen diffract light into a range of directions, schematically shown as two diffracted beams in Fig. 2a. This light is then captured by the first lens, which generates a spatial Fourier transform in its back focal plane^37^. Here, a Fourier filter selectively manipulates each spatial frequency present in the input field and controls their transmission amplitudes and phases. The second lens finally recombines the different spatial frequency components to form an image. Transparent specimens with weak phase disturbances $\varphi \left(x,y\right)$, imprint small, imaginary perturbations on the strong background field:$p\left(x,y\right)={\exp }\,{\exp }\,{[i\varphi \,\left(x,y\right)]}_{\varphi \left(x,y\right)\to 0}\,\approx 1+i\varphi \left(x,y\right)=1+\varphi (x,y) {\exp }\,{\exp }\,\left(i\pi /2\right)$. The principle of operation of phase-contrast microscopy is conveniently recapped in Supplementary Note 1. In the Fourier plane, a filter can be introduced to produce a high contrast image. The filter attenuates the background and imparts it with a *π*/2 phase shift to bring it back in phase with the diffracted foreground light. Whereas this approach is conceptually very simple, in practice the setups for Zernike’s phase contrast imaging necessities careful insertion of multiple filters in the Fourier planes and thus limits its application in many optical microscopes. (Supplementary Fig. S2).

### Design of a nonlocal angular filter for phase contrast imaging

Here, we show that the advances in flat optics can be leveraged to achieve a phase contrast image in a very different way. By inserting a judiciously-designed angular filter directly behind the specimen, we can achieve the same function as the Fourier filter (Fig. 2a). For optimal phase contrast in weakly scattering samples this filter should be engineered to selectively attenuate and impart a *π*/2 phase-shift to the rays that travel near the optical axis. Three significant practical advantages of using an angular filter are immediately obvious. First, such a filter enables an easy change in the focal length of the lenses in a 4 f system without a need to reposition optical components. Second, as no optical manipulations are required in the Fourier plane, only one lens is required to form the image. Third, the optical alignment is greatly simplified since unlike the Fourier filter, which requires accurate positioning, the angular filter can be placed anywhere between the specimen and the objective lens, without any recourse to precise transversal alignment. It is only critical to keep the angular filter orthogonal to the optical axis, but this mono-dimensional requirement is easily accomplished using modern-day opto-mechanics.

To selectively manipulate the paraxial rays, we can employ a metasurface that exhibits a high optical quality factor (*Q*) with a nonlocal response^19,38^. We employ a NLM that supports a guided-mode resonance^39–44^. It is comprised of a surface-relief grating (p = 390 nm) etched into a 240-nm-thick single-mode silicon nitride (Si_3_N_4_) slab waveguide placed on a fused silica substrate (Fig. 2b). To appreciate the wavelength- and angle-dependent light transmission properties of the NLM, it is important to realize that the incident light can follow two distinct pathways through such an optical element. Most of the incident light waves follow a direct transmission pathway through the patterned nitride layer. Their transmission properties follow that of a regular, low-quality-factor (*Q* ≈ 2) Fabry-Perot resonator. However, at selected wavelengths and angles, the grating can resonantly couple the incident light to quasi-guided modes. This can lead to a notable energy storage in the waveguide and a substantial increase in the electric field in the nitride layer. Conversely the presence of the grating causes the guided light to slowly leak out of the structure and in the forward direction it interferes with the directly transmitted light. This interference gives rise to a pronounced dip in the transmission spectrum (Fig. 2c). The amplitude of the grating elements determines the coupling efficiency in/out of the waveguide and thus also the radiative quality factor (*Q*) for the resonances. In our case, we select a grating period of 390 nm to place a resonance at 630 nm for normally-incident light with a transverse magnetic (TM) polarization (the magnetic field is pointing along the grooves of the grating). The grating depth was chosen to be 90 nm to produce a resonance with a quality factor *Q* ≈ 60. This results in a spectrally-narrow resonance with a full-width-at-half-maximum (FWHM) of 11 nm, almost twice the bandwidth (6 nm) of our spectrally-filtered white-light supercontinuum source with which we illuminate our samples.

Figures 2d and 2e show the simulated angle-dependent transmittance and phase-shift on resonance (λ_0_ = 630 nm) and for illumination wavelengths 3 nm above and below it. Here, the phase shift is taken to be the total phase accumulation for light as it propagates from the top to the bottom surface of the NLM. On resonance, we see that our angular filter performs the intended function needed for phase-contrast imaging. A strong reduction of the transmitted light intensity occurs at near-normal incidence (i.e., for paraxial rays) while the non-paraxial rays are virtually unattenuated. The kink in the angle-dependent transmittance spectrum (Fig. 2d) at an angle of 10^o^ shows that a diffraction order opens up, but it does not carry significant power (<5%) for our small-amplitude dielectric grating. The resonance also ensures that the paraxial rays (non-diffracted light) incur a π/2 phase shift with respect to the non-paraxial ones (diffracted light). To ensure that essentially all of the diffracted light from the specimen is transmitted, narrowband angular filtering is necessary. In our case, our chosen grating amplitude of 90 nm achieves an angular FWHM of 2° for our transmission dip. This corresponds to spatial frequencies (*f*_*x*_) as low as 1/18 μm^−1^.

The angle-dependent transmission amplitude and phase both display notable changes as we move away from the resonance wavelength. This provides a unique opportunity that is not available in regular phase contrast imaging; different optical transfer functions can be achieved by simply tuning the illumination wavelength (Supplementary Note 3 and 4). By tuning the illumination wavelength below the resonance (e.g. λ = λ_0_ – 3 nm, blue spectrum), we can reduce the amplitude of the paraxial rays and thereby adjust the relative strength and phases of the background versus the foreground to achieve maximum contrast. Such an adjustment is advantageous for specimens with weaker phase disturbances that diffract less light. This feature allows the optical system with an angular filter to maintain high contrast for a broader range of phase objects than the traditional Zernike approach. When the illumination wavelength is tuned above resonance (e.g. λ = λ_0_ + 3 nm, green spectrum), we can see that the relative phase pickup for the background and foreground is reversed in sign. This makes it possible to capture both positive and negative phase contrast images with the same optical setup by simply tuning the illumination wavelength (see insets to panel e and Supplementary Fig. S4).

### Experimental validation of angle-dependent optical response

**Fig. 3:**
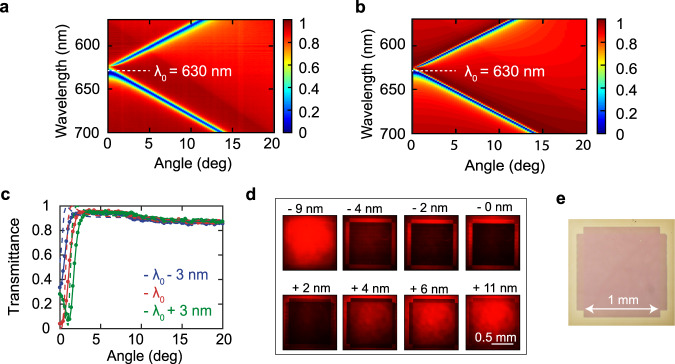
Experimental validation of the angular response of our NLM. **a** Experimental map of the metasurface transmittance versus wavelength and incident angle that reflects the dispersion of the transverse magnetic (TM) mode of the single-mode silicon nitride slab corrugated with the grating. **b** Simulated map of the transmittance that closely matches the experiments in panel **a**. **c** Angle-dependent transmittance of the NLM at three wavelengths near the resonant wavelength λ_0_ = 630 nm. (dashed line: simulation, solid line: experiment) **d** Normal incidence transmission images of the NLM taken at various illumination wavelengths around λ_0_ = 630 nm show the reduced transmission across the entire patterned area on resonance (NKT supercontinuum source, TM polarized). **e** Reflection optical microscope image of the NLM.

We fabricate a large area (1 mm^2^) NLM with electron-beam lithography (Supplementary Fig. S5). We then experimentally determine the dispersive optical properties of our NLM through a series of angular and spectral transmission measurements in a home-built setup. The NLM is illuminated with TM-polarized light of different wavelengths using a halogen lamp. Figure 3a shows the measured transmission maps that highlight the spectral dependence of the angular transmission dip. The measured data closely matches the simulated transmittance shown in Fig. 3b based on the rigorous coupled-wave analysis (RCWA)^38,39^. The good agreement enables us to attribute the reduced transmission feature to the resonant excitation of the transverse magnetic (TM) waveguide mode of the NLM. A detailed comparison of the experimental and simulated transmission spectra shows a small difference in the background transmission, which is attributed to the approximately 4% reflection from the backside of the fused silica substrate.

Figure 3d analyzes the spatial dependence of the transmission through the patterned 1 mm^2^ NLM under the illumination with a collimated (angular spread 0.02°) spectrally-filtered (FWHM = 6 nm) supercontinuum source at different wavelengths. On resonance, the square metasurface appears dark and its outline is clearly defined. We find that 97% of the normally incident (paraxial) light on resonance is blocked and the transmitted intensity increases away from resonance. The uniformity of the image verifies the excellent control over the metasurface grating parameters across the entire area of the device.

### System integration for quantitative phase contrast imaging

**Fig. 4:**
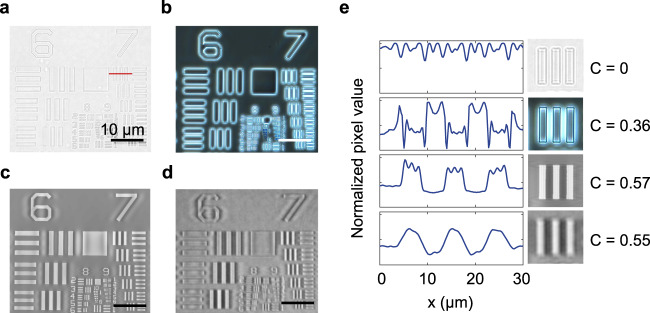
A quantitative comparison of conventional and NLM phase contrast images. **a** Bright field image of a phase-contrast calibration sample. **b** Phase contrast image of the calibration sample recorded with Zernike’s method. **c**, **d**. **c** Simulated, and **d**. experimental phase contrast images taken with the NLM. **e** Line profiles of the bars in group 7 of the calibration sample (indicated by the red line in panel **a**) allow for a quantitative comparison of the different imaging approaches. From top to bottom, the profiles are extracted from panels **a**, **b**, **c**, and **d**, respectively. C represents the contrast values. All images are measured on a polymer USAF target (*n* = 1.5) that has 200 nm thickness.

To quantitatively compare the performance of the conventional Zernike phase-imaging approach to ours, we image a commercially-available US air force phase target with thin transparent elevated polymer patterns. The detailed layout of the optical setups can be seen in Supplementary Fig. 2. For both imaging approaches, we use lenses with the same numerical aperture (NA = 0.45). For the Zernike’s method, a phase annulus is embedded in the objective lens, while for the other method, a standard bright field objective is used. Figure 4a–d show transmission images taken by both techniques. Figure 4e also shows detailed line-cuts of the first element of group 7, featuring phase elements with a line width of 3.71 μm). We compare the contrast for the same spatial frequency (group 7, element 1) to minimize the impact of a possible dependencies of the modulation transfer function of our optical system on the spatial frequency. We adopt Michelson’s definition of contrast for quantitative comparison:

1 $$C({k}_{x})=\frac{{I}_{{\max }}-{I}_{{\min }}}{{I}_{{\max }}+{I}_{{\min }}}$$

As expected, we see close to zero contrast in the bright field image. In the zoomed-in picture, most of the contrast we see comes from the slight chromatic dispersion of the phase sample. It is well-established that zero contrast is expected for phase objects that are perfectly in focus while being imaged with a high NA imaging system and monochromatic light source^37^. Compared to bright field imaging, the phase contrast microscope provides much higher contrast in the contours of the features. However, its line profile does not faithfully reproduce the sample topology due to the lack of accurate spatial frequency filtering. In reality, the phase annulus that attenuates and phase-shifts the background in most phase contrast microscopes is intentionally engineered to be (3-5 times) wider than needed (the width of the condenser annulus ring) to facilitate the manual alignment and reduce pinhole diffraction (Supplementary Figure 2b) and has a fixed amount of attenuation that may not be optimal for the sample under investigation. The resulting non-optimal spatial filtering produces a set of well-studied artifacts, such as halo and shade-off^45,46^. Whereas Zernike’s method can produce satisfying and insightful images, this limitation makes it challenging to perform quantitative phase measurements.

Figures 4c and 4d show the simulated and measured images obtained with our NLM. The high *Q* resonance of our device allows for precise filtering at target angles. As a result, the NLM method can be successfully applied at lower spatial frequencies (group 6 elements in the phase target) than typically accessible in commercial phase contrast microscopes due to the relatively large phase annulus. In both simulated and measured NLM phase contrast images, we see small ripple-like patterns around features with a 16 μm period. This could be mathematically explained by the convolutional theory, in which the ripples arise from convolution with the Fourier transform of the angular transmittance transfer function (Supplementary Fig. S7). This artifact may be eliminated with the use of an isotropic two-dimensional NLM^47^. In Supplementary Fig. S8, we provide a zoomed-in image of the smaller features. Features as small as 780 nm (Group 9, element 3) can be resolved, close to the diffraction limit at the chosen NA (0.45) and wavelength. This demonstrates that the resolution of this imaging method is limited by the NA of the lenses and pixel sizes, not by the NLM. This is because the NLM remains highly transparent until large incident angles (T = 75% at 75°, equivalent to NA = 0.97 shown in Supplementary Fig. S9 in Supplementary Note 8). Thus, the insertion of the NLM barely introduces degradation of the modulation transfer function (MTF) of the imaging system at high spatial frequencies and does not affect the final image resolution.

**Fig. 5:**
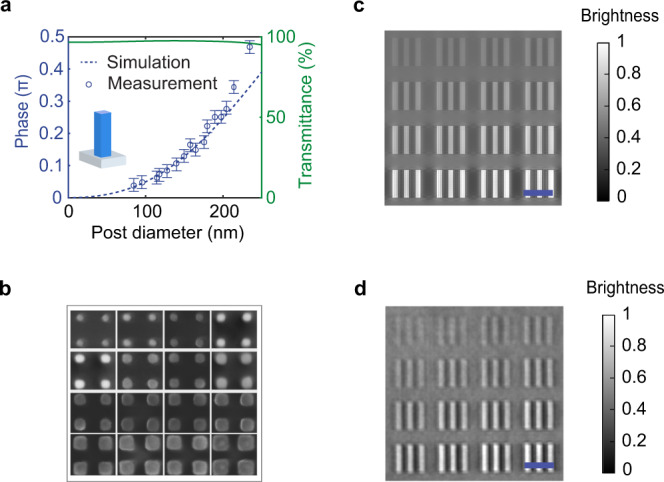
Quantitative phase retrieval using an NLM. **a** Transmission and phase delay from 2D periodic arrays of Si_3_N_4_ nanorods with heights of *h* = 230 nm and lattice constant *a* = 360 nm. The inset shows the structure of a single cell. The error bars show a reference of $\pm 0.02\pi$ phase difference with respect to the measured phase. **b** SEM image of different area of the fabricated metasurface. **c** Simulated NLM phase contrast image. **d** Measured NLM phase contrast image. Scale bar corresponds to 20 µm.

To demonstrate the versatility our phase imaging approach, we illustrate its applicability to the rapidly growing field of metasurface optics^40–48^. Metasurfaces are constructed from dense arrays of nanostructures and are capable of manipulating the light field in new ways that go beyond the capabilities of conventional optical elements. Most metasurfaces accomplish this feat by controlling the local phase of scattered light and are termed local metasurfaces (LMs). Thus, their optical characterization critically relies on an accurate measurement of the phase profile of fabricated elements. Figure 5a shows how we can apply the developed technique to image a typical LM patterned into a Si_3_N_4_ layer on a fused silica substrate. This LM is comprised of a dense forest of nitride nano-posts that are arranged in a square lattice with a lattice constant *a* = 360 nm. The side length of the nano-post is varied from area to area (Fig. 5b) to achieve phase pickups in the range from 0.04 $\pi$ to 0.4 $\pi$. As the first step, we calibrate the NLM contrast using the USAF phase target (Supplementary Fig. S9). Then, we apply NLM phase contrast imaging to the LM to visualize and quantify the phase delays in different areas, as shown in Fig. 5a, c. The recovered phase profile agrees well with the simulated result, particularly in the weak phase regime (<0.4 $\pi$) where the accuracy is better than 0.02 $\pi$. This shows that NLM contrast shows high accuracy in obtaining a quantitative phase profile.

## Discussion

Our results show that inserting a nonlocal metasurface in the optical path of a regular optical microscope enables quantitative phase contrast imaging with a range of unique imaging benefits over traditional phase contrast imaging. In our experiments we demonstrated the operating principle with linearly polarized illumination of an anisotropic NLM with a supercontinuum source. However, it should be noted that polarization-independent NLMs can be designed as well and that less expensive super luminescent diodes have sufficiently narrow bandwidths for this type of imaging. Unlike the conventional implementation of Zernike’s phase contrast, our method does not require access to the back focal plane and therefore enables the development of more compact imaging systems. At a high level, this work adds to the rapidly growing interest in using flat optics for analog optical computing and image processing.

## Methods

### Fabrication

We deposit silicon nitride film on a fused silica substrate by plasma-enhanced chemical vapor deposition (PECVD). The thickness and the optical constants of the film are characterized by ellipsometry (Woollam, M2000 Spectroscopic). The grating patterns are defined by electron beam lithography (JEOL JBX-6300FS) followed by reactive ion etching (Oxford, PlasmaPro 80). More details of the fabrication can be found in Supplemental Note 5.

### Characterization

The final dimensions of the structure are measured using scanning electron microscopy (FEI Magellan 400 XHR) and an atomic force microscope (Park XE-70). For the optical characterization, we use a halogen lamp for the broadband light illumination in a home-built angle-dependent transmission measurement setup. The transmittance spectrum is captured by a commercial spectrometer (SpectraPro, Acton 2300i). We use two rigorous coupled-wave analysis (RCWA) programs for the numerical simulations. For in-plane incidence cases, we use a freely-released Matlab code developed by Dr. Zhao Bo in Prof. Zhuomin Zhang’s group at Georgia Tech University. For conical illumination cases, we use the RETICOLO freeware^48^.

#### Cells source

The onion cells used in Fig. 1b was obtained from an onion purchased from a grocery store. The human osteosarcoma cells U-2 OS (ATCC HTB-96) used was obtained from ATCC.

### Reporting summary

Further information on research design is available in the Nature Research Reporting Summary linked to this article.

## Supplementary information

Supplementary Information

Reporting Summary

## Data Availability

The data that support the plots within this paper and other finding of this study are available from the corresponding author upon request.
