# Supplementary material for: Quantitative phase contrast imaging with a nonlocal angle-selective metasurface
Source: Nat Commun. 2022 Dec 21;13:7848. doi: 10.1038/s41467-022-34197-6 (PMC9772391; doi:10.1038/s41467-022-34197-6)
Supplement: Supplementary file 1 — Supplementary Information [file 41467_2022_34197_MOESM1_ESM.pdf]

Supplementary Information

Quantitative Phase Contrast Imaging with a Nonlocal Angle-selective Metasurface

Anqi Ji[1], Jung-Hwan Song[1], Qitong Li[1], Fenghao Xu[1], Ching-Ting Tsai[2], Richard C. Tiberio[3], Bianxiao Cui[2], Philippe Lalanne[4], Pieter G. Kik[5], David A. B. Miller[6], Mark L. Brongersma[1*]

[1] Geballe Laboratory for Advanced Materials, Stanford University, Stanford, CA94305, USA.

[2] Department of Chemistry, Stanford University School of Medicine, Stanford, CA94305, USA.

[3] Stanford Nano Shared Facilities, Stanford University, Stanford, CA94305, USA.

[4] LP2N, CNRS, University of Bordeaux, 33400 Talence, France.

[5] CREOL, The College of Optics and Photonics, University of Central Florida, Orlando, FL32816, USA.

[6] Department of Electrical Engineering, Stanford University, Stanford, CA94305, USA.

[*] Correspondence and requests for materials should be addressed to Mark L. Brongersma (email: brongersma@stanford.edu).

The supplementary information contains text and eight figures in support of the main body of the text. It discusses specific topics:

S1. Mathematical description of general phase contrast imaging
S2. Optical systems for phase object imaging.
S3. NLM phase contrast simulation algorithm.
S4. Operating wavelength of NLM phase contrast imaging
S5. Fabrication of an NLM.
S6. Artifact analysis for NLM contrast imaging.
S7. Angle dependence transmittance of GMR for large oblique incident angles.
S8. Calibration for quantitative phase retrieval.

Supplementary Note 1: Mathematical description of general phase contrast imaging.

To understand the principle of phase contrast imaging, we go through a case of one-dimensional phase grating with weak undulation (Fig. S1a)[1,5]. The phase profile of the grating can be expressed as

$$\varphi(x) = c\sin(f_x x) \tag{S6}$$

Here, $f_x = 0.1\,um^{-1}$ (period = 10 um), while $c = 0.25\,rad$. When a normal incidence plane wave ($E_0 = 1$) passes through a phase sample, the electric field of the transmitted light can be approximated when the phase undulation is small (c < $\pi/3$).

$$E_{in}(x,y) = E_0\exp[i\varphi(x,y)] \approx 1 + i\,\mathrm{csin}(f_x x) \tag{S7}$$

The electric field can be further decomposed into Fourier series to obtain the Fourier coefficient of different spatial frequencies.

$$E_{in}(x,y) = 1 + c_{1,0}\exp(if_x x) + c_{-1,0}\exp(-if_x x) \tag{S8}$$

$c_{m,n}$ is the Fourier coefficient of the mth diffraction order in x direction and the nth order in y direction. In Fig. S5b, we show the amplitude of these coefficients ($|c_{1,0}| = |c_{-1,0}| = 0.125$, $|c_{0,0}| = 1$).

To enhance the phase contrast, the phase of the DC component needs to be advanced or delayed 90 degrees to be in phase of the diffracted orders. The amplitude of the DC component should also be reduced to further boost the contrast. Assuming the DC component is 90 degree phase advanced and reduced to amplitude *a*, the electric field becomes,

$$E_{out}(x,y) = ia + i\,\mathrm{csin}(f_x x) = \mathrm{i}(a + \mathrm{csin}(f_x x)) \tag{S9}$$

The corresponding contrast becomes

$$C = \frac{I_{max} - I_{min}}{I_{max} + I_{min}} = \frac{(a+c)^2 - (a-c)^2}{(a+c)^2 + (a-c)^2} \tag{S10}$$

The contrast is maximized (C = 1) when $I_{min} = (a-c)^2 = 0$.

Figure S1c shows the phase contrast images when DC attenuation a is above, equal to and below the amplitude of the phase grating (c = 0.25). When DC attenuation is larger than c (Fig. S1c left), the image shows a smaller contrast than when the DC attenuation is equal to c (Fig. S1b middle). When the DC component is over reduced (Fig. S1c right), spatial frequency of the image doubles compared to the original phase profile. This effect can be calculated by calculating the intensity profile of the image,

$$I_{a=0}(x) = \ |c\sin(f_x x)\,|^2 = \frac{c^2}{4}[1-\cos(2f_x x)\,] \tag{S11}$$

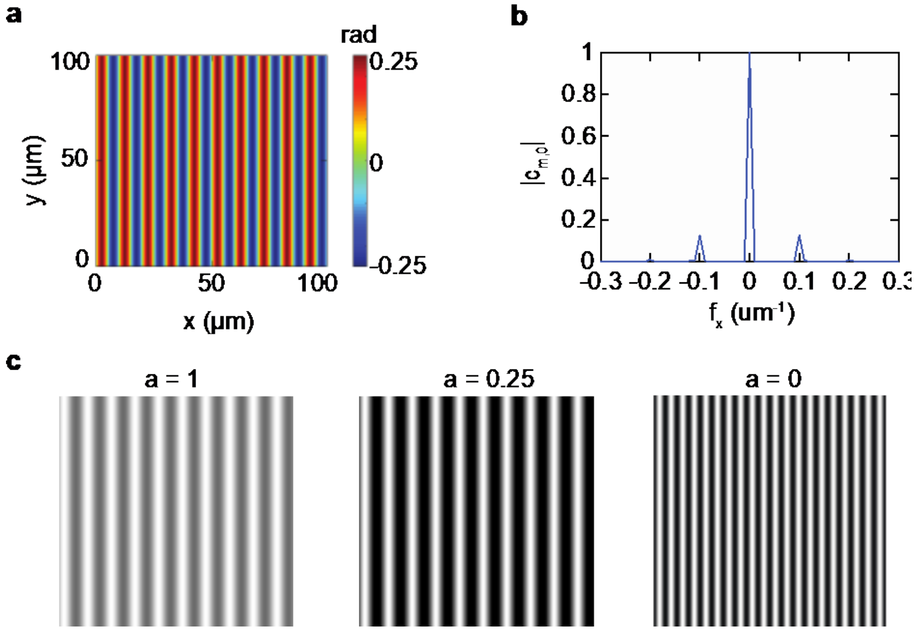

Figure S1. Operating wavelength for maximized phase contrast. **a.** The phase profile of a phase grating. **b.** The amplitude of Fourier coefficients the phase grating. **c.** The simulated phase contrast images when the DC reduction above (a = 1), equal to (a = 0.25) and less than (a = 0)) the sum of coefficients of first diffraction order.

Supplementary Note 2: Optical systems for phase object imaging.

In Fig. 3, we showed the images of a phase specimen in bright field, Zernike's phase contrast[1] and NLM phase configuration. In this section, we explain the optical layout for these three configurations. In a bright field setup shown in Fig. S1a, a phase sample is placed in the specimen plane. Both the unscattered light (DC) and the scattered light from the phase sample are collected through an objective lens, resulting in weak contrast.

As shown in Fig. S1b, Zernike's phase contrast imaging uses a set of condenser annulus (D-C Phase Ring, Module PH-1 in NI-CUD UNIVERSAL CONDENSER DRY-NC) and a phase plate (Ph1 Ring in CFI SUPER PLAN FLUOR ELWD 20XC ADM) to selectively modulate the DC component. The illumination angle is first defined by a condenser annulus placed in the Fourier plane below the specimen. A corresponding phase plate is located in the Fourier plane inside the objective. The unscattered light (DC component) from the phase object later passes through a ring in the phase plate, experiencing both absorption and 90° phase attenuation. After the phase plate, all angular components focus and interfere on the image plane, forming a phase contrast image.

Compared to Zernike's method, the nonlocal metasurface (NLM) phase contrast uses a greatly simplified setup, shown in Fig. S1c. An NLM is placed between the objective lens and the specimen (it can also be placed near the detector). The NLM directly attenuates the amplitude and phase of the DC component without creating a Fourier plane. The system simplification lead to three major advantages of NLM phase contrast imaging compared to Zernike's method. First, the alignment process of the system is shortened. In Zernike's setup, a new matching phase plate and condenser are needed for every objective magnification. Each time, the alignment of phase plate and the condenser annulus needs to be confirmed in the Fourier plane, as shown in the inset of Fig. S1b. This is inconvenient in microscopy studies where multiple sample magnifications are necessary. Second, a more precise DC filtering can be achieved with NLM phase contrast. As shown in the inset image of Fig S1b, the phase ring does not exactly overlay with the phase annulus in the Fourier plane. The width of a phase ring (1) is generally 3-5 times wider than that of the condenser annulus (2) for the purpose manual alignment and reduction of scattering from small pinholes. The inaccurate filtering results in the missing of low spatial frequencies in the final image. As NLM phase contrast can be easily designed with very high Q, the angular filtering can be performed in a much more precise way. Last, the cost of the system integration is

reduced. In the Zernike's method, the phase plate has to be placed in the Fourier plane which is commonly inside an objective. When a new phase plate is needed, a new objective has to be purchased. The NLM phase contrast method eliminates this need since a Fourier plane is not required.

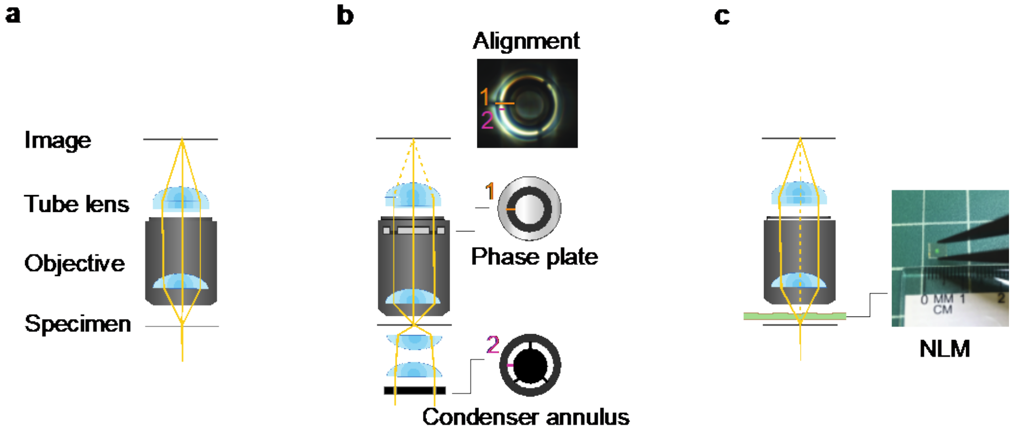

Figure S2. Optical layout of different imaging methods used for phase objects. **a.** Bright field imaging. **b.** Zernike's phase contrast imaging. **c.** Nonlocal metasurface (NLM) phase contrast imaging. Solid lines show the original light scattered or transmitted from the specimen. Dash lines show the light modulated by a phase plate or an NLM.

Supplementary Note 3: NLM phase contrast simulation algorithm.

Figure S3 shows the algorithm used in the NLM phase contrast image simulation pipeline. We start with a given phase profile $\varphi(x, y)$. When a normal incidence plane wave ($E_0 = 1$) passes through a phase sample, the electric field of the transmitted light is expressed as:

$$E_{in}(x, y) = E_0 \exp\left[i\varphi(x, y)\right] \tag{S1}$$

This is the electric field spatial distribution directly before the NLM. Then, we obtain the angular spectrum ($\mathrm{c}(k_x, k_y)$) of the electric field through Fourier transform.

$$\mathrm{c}(k_x, k_y) = FT\{E_{in}(x, y)\} \tag{S2}$$

Meanwhile, we calculate the angle dependent transmission coefficient $t(k_x, k_y)$ of an NLM using RCWA. Later, we apply the angular transmission spectrum to the Fourier coefficient map through element-wise multiplication.

$$c'(k_x, k_y) = \mathrm{c}(k_x, k_y)t(k_x, k_y) \tag{S3}$$

The electric field spatial distribution directly after the NLM can be calculated by inverse Fourier transform.

$$E_{out}(x, y) = FT^{-1}\{c'(k_x, k_y)\} \tag{S4}$$

Finally, the intensity map (image) of the NLM contrast image is obtained by taking the square of the electric field.

$$I_{out}(x, y) = |E_{out}(x, y)|^2 \tag{S5}$$

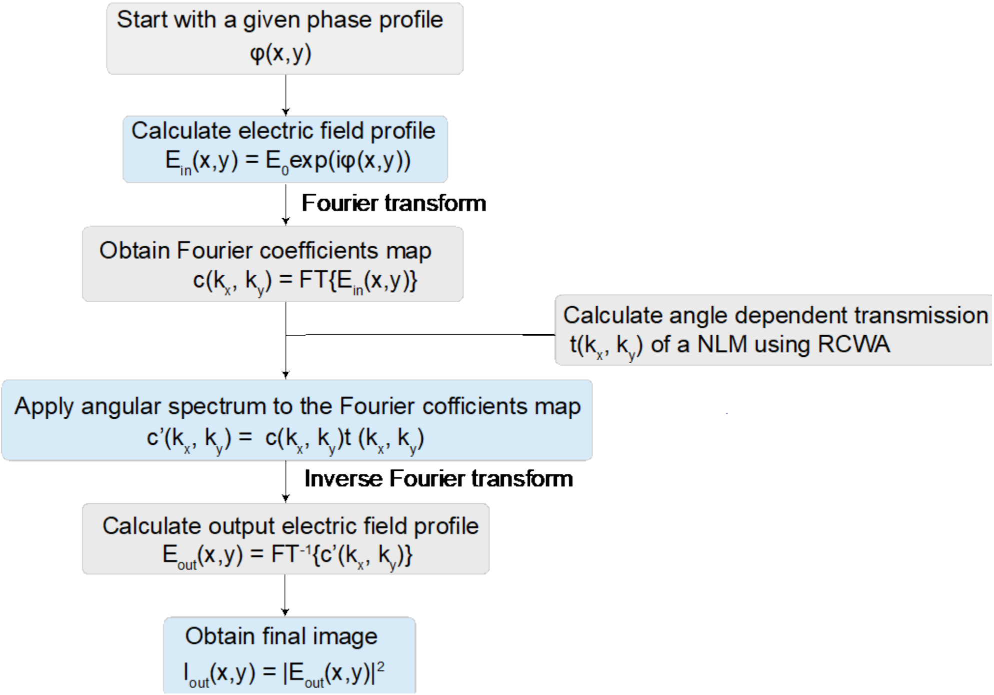

Figure S3. NLM contrast image simulation pipeline.

Supplementary Note 4: Operating wavelength of NLM phase contrast imaging

As shown in Fig. 2, the NLM phase contrast of a sample can be changed by tuning the illumination wavelength. In this section, we discuss how to determine the operation wavelength for NLM phase contrast image.

In Supplementary Note 1, we show that the phase contrast can be tuned by adjusting the DC background reduction factor a. The more the operating wavelength is close to the resonant wavelength, the more DC background is reduced. Therefore, to obtain the maximized phase contrast, the illumination wavelength needs to be adjusted such that the DC attenuation is equal or larger than the DC phase grating amplitude, while in reality the DC phase grating amplitude varies depending on the index and height variation of the phase object.

While this condition is satisfied, two types of phase contrast can be achieved by advancing (negative contrast) or delaying (positive contrast) the DC component phase. In Fig. S6, we show the simulated NLM phase contrast of the same phase grating when operating at different wavelengths. The blue box region shows when the DC attenuation is over-reduced ($a<c$). Outside this region, when the illumination wavelength λ is below/above resonant wavelength λ_0, the image has positive/negative contrast. The inset images show the appearance of positive contrast and negative contrast images obtained through experiments. The positive and negative contrast images are obtained when the λ is 6 nm below and above λ_0, respectively. When the illumination wavelength gets further and further from the resonance, the contrast of the image approaches zero as NLM only attenuates the amplitude and phase of the DC component around resonance.

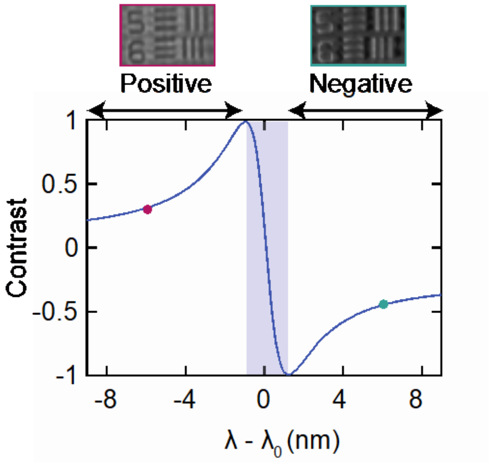

Figure S4. Tuning wavelength to achieve contrast and negative contrast. The insets show the positive and negative contrast experimentally obtained at different wavelengths.

Supplementary Note 5: Fabrication of an NLM.

A 525 um thick fused silica substrate is first cleaned (Fig. S2a) by a standard RCA process[2] to remove the organic and ionic contaminants. A 236 nm-thick silicon nitride film was deposited using plasma-enhanced chemical vapor deposition (PECVD) on the substrate. For the patterning, we used positive-toned CSAR 62 (9%) as the resist. A 1 mm^2 area NLM was patterned using electron-beam lithography (JEOL JBX-6300FS). To avoid stitching error between writing fields, multiple pass exposure (MPE) method[3,4] was applied. After developing the sample in Xylene, 90 nm thick nitride was etched away using a mixture of carbon tetrafluoride, fluoroform and argon plasma (Oxford 80 reactive ion etcher). During dry etching, the remained CSAR serves as a hard mask. After the dry etching, the CSAR mask was removed by soaking in acetone overnight. The final dimensions of the device were confirmed through scanning electron microscopy (SEM: FEI Magellan 400 XHR) and atomic force microscopy (AFM: Park XE-70) imaging. The optical constant of the nitride film is characterized using ellipsometry (Fig. S3). At target wavelength $\lambda = 630\ nm$, the refractive index of the nitride is 2.02 while the extinction coefficient is nearly zero.

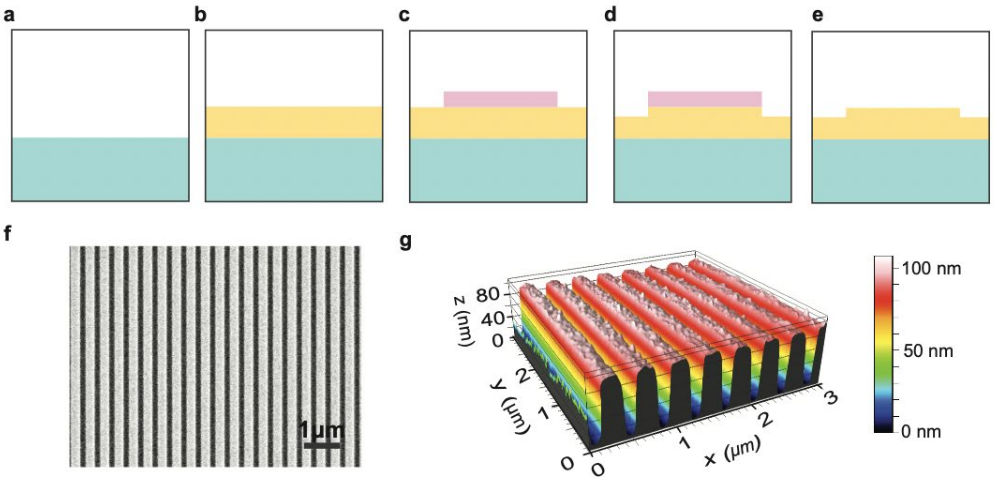

Figure S5. Fabrication of an NLM. **a.** Standard RCA cleaning of the silica substrate (shown in green). **b.** Deposition of 236-nm-thick Si_3N_4 using PECVD (shown in yellow). **c.** Electron beam lithography on 200-nm-thick CSAR resist (shown in pink). **d.** 90 nm plasma etching into Si_3N_4 film. **e.** Removal of resist. **f.** SEM image of the fabricated sample. **g.** AFM image for the height variation of the sample.

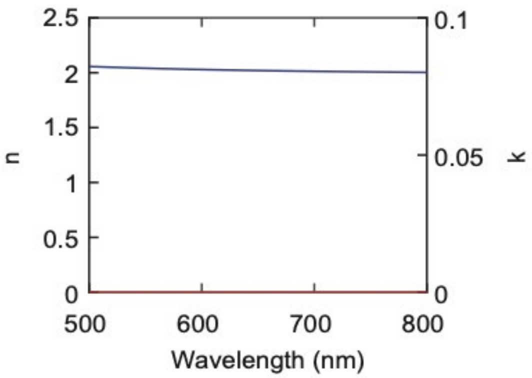

Figure S6. Measured refractive index of Si_3N_4 film from ellipsometry. Blue line shows the refractive index *n*, while the red line shows the extinction coefficient *k*.

Supplementary 6: Artifact analysis for NLM contrast imaging.

In Fig. 4 c and d, we observe tiny ripples around isolated features. In this section, we analyze the origin of such ripples. Figure S7a shows the image and the cross section of ripples. The ripples appear in 16 μm from the center of the element. The angular transmittance map of the corresponding NLM is shown in Fig. S7b. The origin of the 16 μm periodicity ripples is associated with the elongated NLM transmission dip at $f_x = 0.06$ μm^{-1}. The effect is analogous to the diffraction phenomenon from a narrow slit in the space domain[5]. According to convolution theorem, multiplication in the Fourier domain is equivalent to convolution in the space domain. A multiplication with a slit at $f_x = 0.06$ μm^{-1} results in a convolution of $1/f_x$ periodicity in the space domain.

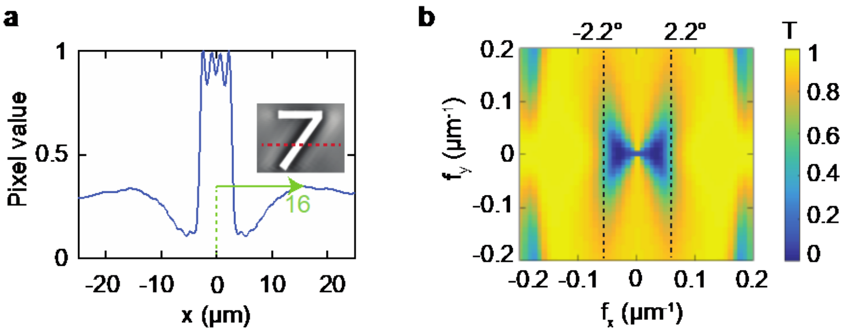

Figure S7. Image artifact analysis. **a**. Cross section (on the red line in the inset) of a simulated NLM phase contrast image (h = 200 nm, $\lambda = \lambda_0 + 3\ nm$). The inset image is copied from Fig. S7a. The contrast of the inset image is digitally boosted to highlight the artifact. **b.** Simulated NLM angular transmittance map. The image artifact with periodicity 16 μm originates from the elongated NLM transmission dip at spatial frequency of 0.06 μm^{-1} (2.2°) in the y direction.

Supplementary Note 7: Angle dependence transmittance of GMR for large oblique incident angles.

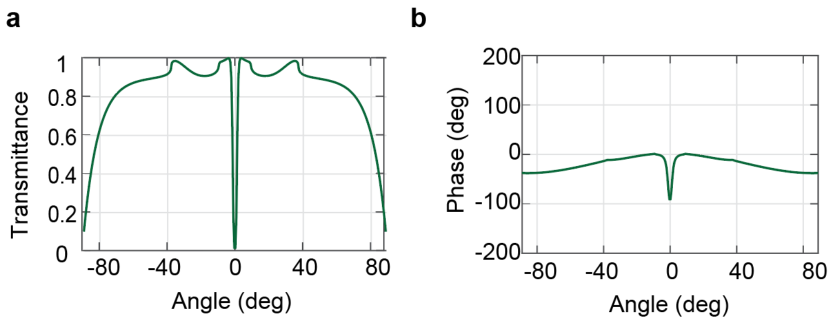

Figure S8. Simulated angular transmittance (**a**) and phase shift (**b**) of the NLM at high oblique angles.

Supplementary Note 8: Calibration for quantitative phase retrieval.

Besides the NLM, the actual contrast can be also influenced by the modulation transfer function of the optical system and the illumination spectrum. In order to retrieve the phase quantitatively, we used a commercial phase target (Quantitative Phase Target from Benchmark Technology) to calibrate the NLM. In the phase target, polymer USAF resolution target of 7 different heights (50 nm to 350 nm) is defined by electron beam lithography and then transferred onto a glass substrate. The refractive index of the polymer is offered by the vendor (n = 1.52) and the height of the phase target (h) is measured with AFM.

We show the simulated (Fig. S9a) and measured phase contrast images (Fig. S9b) of different heights. The illumination center wavelength is 3 nm above the resonate wavelength ($\lambda = \lambda_0 + 3\ nm$). In the simulation, the illumination has a 6 nm FWHM with Gaussian distribution, while in the measurement a super continuum source (NKT) with a 6 nm FWHM was used. As the height of the polymer pattern increases, the phase contrast increases. In the inset of Fig. S8b, we show a zoomed image of the smaller features (resolution target group 8-10). Features as small as 780 nm (group 9 element 3) can be resolved in the NLM contrast method, indicating that the NLM contrast method does not reduce the resolution of the optical system. The resolution of the system is ultimately limited by the illumination wavelength, numerical aperture (NA = 0.45) and the pixel sizes.

Fig. S9c shows a quantitative comparison of the image contrast between the simulation and the experiment. In the simulation, as the bandwidth of the light source increases, the overall contrast reduces since the final image becomes a weighted sum of a narrowband of monochromatic cases. The experimentally measured contrast is slightly larger than the simulation with thinner samples (h < 200 nm), and larger with thicker samples (h > 200 nm). Although we have not quantitively analyzed the origin of the difference, we suspect that the small gap is due to the subtle difference between the simulated illumination profile and the modulation transfer function of the optical system and the experiments.

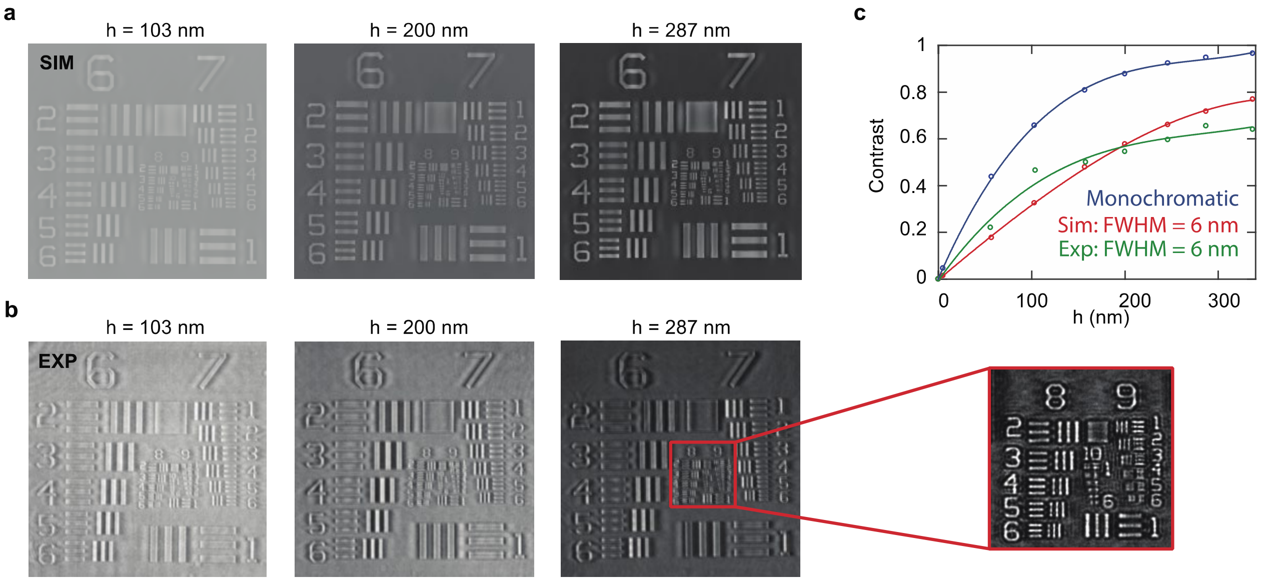

Figure S9. Calibration for quantitative phase retrieval. **a**. Simulation of NLM phase contrast image for samples of different height ($\lambda = \lambda_0 + 3\ nm$, FWHM = 6 nm). **b**. Measured NLM phase contrast images for samples of different height ($\lambda = \lambda_0 + 3\ nm$, FWHM = 6 nm). The inset is a zoomed image of the smaller features (resolution target group 8-10) taken with a magnification lens in front of the detector. **c**. Relationship between NLM contrast and sample height.

Supplementary References

S1. Zernike, F. Phase contrast, a new method for the microscopic observation of transparent objects part II. *Physica* **9**, 974–986 (1942).

S2. Kern, W. The Evolution of Silicon Wafer Cleaning Technology. *J. Electrochem. Soc.* **137**, 1887–1892 (1990).

S3. Heusinger, M., Banasch, M. & Zeitner, U. D. Rowland ghost suppression in high efficiency spectrometer gratings fabricated by e-beam lithography. *Opt. Express* **25**, 6182–6191 (2017).

S4. Albert, J. *et al.* Minimization of phase errors in long fiber Bragg grating phase masks made using electron beam lithography. *IEEE Photonics Technol. Lett.* **8**, 1334–1336 (1996).

S5. Goodman, J. W. *Introduction to Fourier optics*. (Roberts and Company Publishers, 2005).
